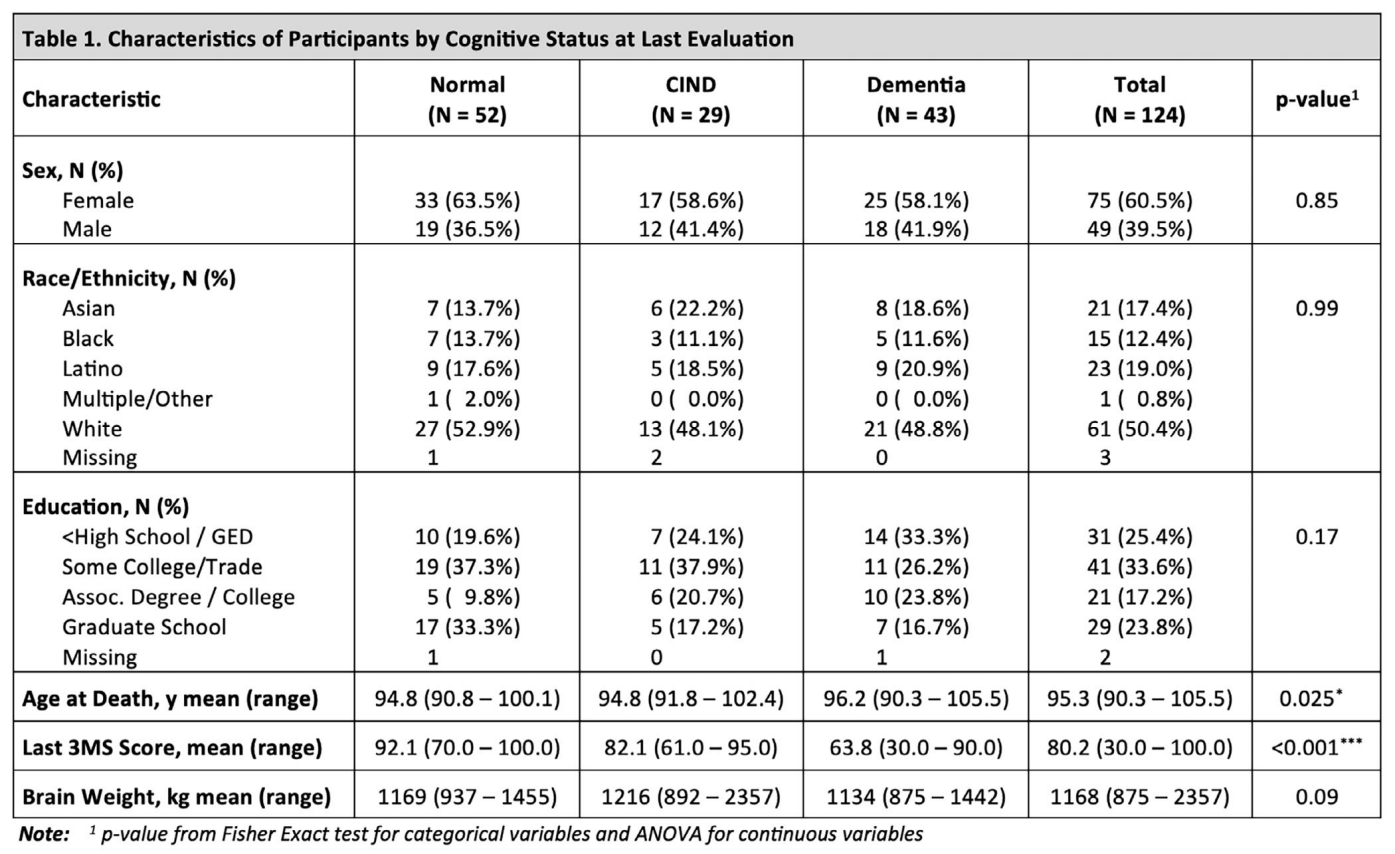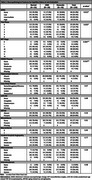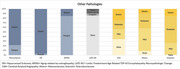# Neuropathology in the LifeAfter90 Study: 2025 update on an Ethnically Diverse Cohort Study of Oldest‐Old

**DOI:** 10.1002/alz70855_106833

**Published:** 2025-12-24

**Authors:** Brittany N Dugger, Lee‐Way Jin, Viharkumar Patel, Melanie N. Luu, Madely Martinez Pamatz, Charles Decarli, Paola Gilsanz, Dan M. Mungas, Claudia H. Kawas, María M. M. Corrada, Rachel A. Whitmer

**Affiliations:** ^1^ University of California, Davis, Sacramento, CA, USA; ^2^ University of California, Davis, Davis, CA, USA; ^3^ Kaiser Permanente Northern California Division of Research, Pleasanton, CA, USA; ^4^ University of California Irvine, Irvine, CA, USA; ^5^ University of California, Irvine, Irvine, CA, USA

## Abstract

**Background:**

Examining the neuropathology of the oldest‐old has significantly advanced our understanding of the multiple etiologies in very late life. Most studies, however, have included mostly White decedents with limited ethnoracial diversity. Our goal was to characterize neuropathology in a cohort of ethnically and racially diverse oldest‐old decedents.

**Method:**

The LifeAfter90 study is an ongoing cohort study of Kaiser Permanente Northern California members, aged 90+ with targeted recruitment of individuals across different racial/ethnic groups with no prior diagnosis of dementia in their medical record. Interviews and cognitive assessments occur approximately every 6 months. Brain donation was available to all interested consenting participants. Neuropathology was assessed using National Alzheimer's Coordinating Center Neuropathology forms and NIA‐AA guidelines for diagnoses.

**Result:**

As of January 2025, 390 participants (34%) had enrolled in autopsy (22% Asian, 20% African American, 18% Latino, 9% Multiracial/Other, and 33% White). Of the 390 participants, 124 had died and neuropathological evaluations completed. The mean age of death was 95 years (range 90‐105), 75 (60%) were female, 21 Asian, 15 Black, 23 Latino, 61 White, and 1 Native American (Table 1). At final clinical exam, 43 participants had dementia (35%), 29 had cognitive impairment without dementia (23%), and 52 had normal cognition (41%). Alzheimer disease (AD) and vascular neuropathologies were the most frequent (Table 2). 79% of participants had at least low likelihood of AD and all but one had neurofibrillary tangles (NFT). However, the most severe level of AD, NFT, and plaques were infrequent. For vascular pathologies, 73% had moderate/severe arteriolosclerosis, 42% moderate/severe atherosclerosis, and 23% 1+ microinfarct. Neurodegenerative pathologies other than AD were less common: 32% had Lewy bodies (6 cases having diffuse type), 24% LATE‐NC, and 4 cases hippocampal sclerosis. Frequencies of other pathologies are shown within the Figure. Cognitive status was associated with the presence of AD neuropathologies, but not other neuropathological changes.

**Conclusion:**

This ethnoracially diverse cohort of oldest‐old individuals reveal numerous brain pathologies are present with advanced age, with AD and select vascular pathologies being the most common. This shows that as in younger cohorts, AD neuropathologies are an important driver of cognitive impairment.